# Type I Ureteral Triplication in an Adult Associated With an Obstructed Extravesicular Megaureter Surgically Managed With Partial Nephrectomy

**DOI:** 10.7759/cureus.51864

**Published:** 2024-01-08

**Authors:** Amanda E Sion, Courtney McClure, Todd Campbell

**Affiliations:** 1 Urology, Ascension Macomb-Oakland Hospital, Detroit, USA

**Keywords:** prostatic ureter, adult, weigert-meyer law, partial nephrectomy, extravesicular ureter, type i triplicated ureter

## Abstract

A complete ureteral triplication is a rare congenital urinary tract anomaly that typically presents in childhood. This is an exceedingly rare case of an adult male presenting with right pyelonephritis and flank pain who was subsequently diagnosed with a right type I ureteral triplication associated with an obstructed megaureter inserted into the prostatic urethra. This patient underwent a right partial nephrectomy to remove the dilated and non-functional upper renal segment leaving behind a blind ending ureteral remnant. Following partial nephrectomy, the patient's flank pain and recurrent urinary tract infections resolved despite persistent dilation of the ureteral remnant. While a standard method of surgical management for a triplicated ureter has not been well established in the adult population, in this case, partial nephrectomy has demonstrated efficacy while avoiding more invasive procedures.

## Introduction

Ureteral triplication is a rare congenital urinary tract anomaly first described in English literature by Lau and Henline in 1931 [1]. In 1946, Smith classified triplicate ureters into four categories to distinguish a complete triplication from other forms [2]. A complete triplication is defined as three separate ureters and ureteral orifices with no connection between the ureters. This is in comparison to a trifid ureter where the ureters join prior to reaching the bladder with only one ureteral orifice, an incomplete triple ureter with a bifurcation of one ureter, and a double ureter with an inverse Y duplication of one ureter. Triplicated ureters are typically diagnosed in the pediatric population and present with recurrent urinary tract infections, incontinence, flank pain, and gross hematuria. The most common urologic abnormalities associated with ureteral triplication are contralateral duplications in 37%, ureteral ectopia in 28%, and renal dysplasia in 8% [3].

We present a case of a 42-year-old male with right flank pain and pyelonephritis in the setting of complete triplication of the right ureter associated with a blind-ending ectopic megaureter inserting into the prostatic urethra associated with a nonfunctioning severely hydronephrotic right upper pole. In adult English literature to date, there have been no reported cases of the management of obstructed ectopic megaureters in a type I triplicate system.

## Case presentation

The patient is a 42-year-old male with a history of kidney stones, complete left ureteral duplication, and complete triplication of the right ureter associated with right upper pole obstruction and hydronephrosis. The patient had numerous hospital admissions dating back to 2008 for periumbilical pain radiating to his right flank with nausea and vomiting. A concurrent gastrointestinal work-up revealed gastroesophageal reflux disease (GERD) secondary to a hiatal hernia and recurrent pancreatitis. Despite recommendations, he did not establish outpatient care with urology.

**Figure 1:**
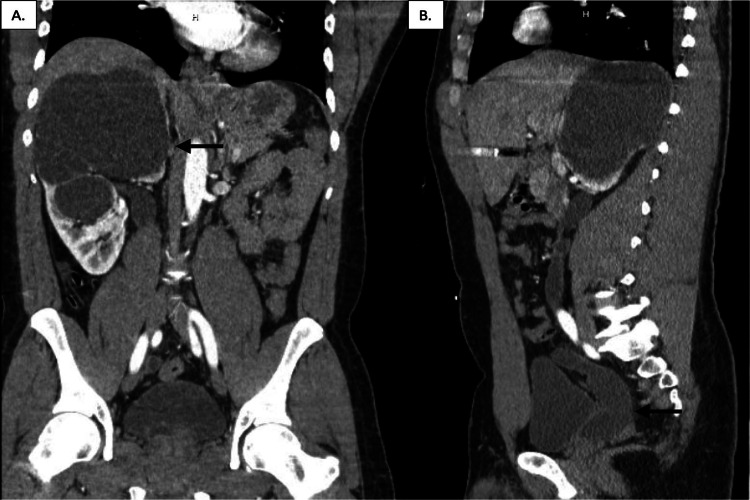
CT abdomen and pelvis without contrast prior to surgery (A) Coronal view of the right kidney revealing severe hydronephrosis of the right kidney with cystic changes. Notice the dilation of the ureter from the upper pole indicated by the black arrow. (B) Sagittal view of severely hydronephrotic right ureter inserting into the posterior prostatic urethra (black arrow).

He presented to the hospital in 2018 with complaints of worsening right flank pain and intermittent gross hematuria. A CT abdomen and pelvis were obtained re-demonstrating a completely triplicated right ureter with severe upper pole hydronephrosis, minimal renal parenchyma, and a megaureter with an ectopic insertion into the prostatic urethra (Figure 1). The patient was taken for cystoscopy with retrograde pyelograms. The left ureter, with known duplication, had a medial ureteral orifice that corresponded to the left upper pole consistent with the Weigert-Meyer law of ureteral duplication. In contrast, the right medial ureteral orifice was associated with the right lower pole. Despite extensive efforts only two ureteral orifices were identified, neither of which demonstrated hydronephrosis. A transurethral incision of the bladder neck and proximal prostatic urethra was made on the right side in an attempt to identify the third ureteral orifices; however, only normal prostatic tissue was seen and the case was aborted. Interventional radiology then placed a percutaneous nephrostomy tube in the right upper pole draining 1400cc of urine. Multiple attempts were made to pass an antegrade wire without success, a nephrostogram was performed and it was concluded the distal ureter was blind ending at the level of the prostatic urethra.

The patient underwent a laparoscopic partial right nephrectomy in 2019 which was converted to an open partial nephrectomy due to adhesions from a past exploratory laparotomy after an abdominal gunshot wound. Three ureters were identified during the procedure and the upper pole was floppy and did not appear to contain much parenchyma which was later confirmed with pathology. The upper pole of the kidney was removed leaving the dilated upper pole ureter with a blind end proximally. The surgery was carried out without complication and he had an uneventful recovery.

**Figure 2:**
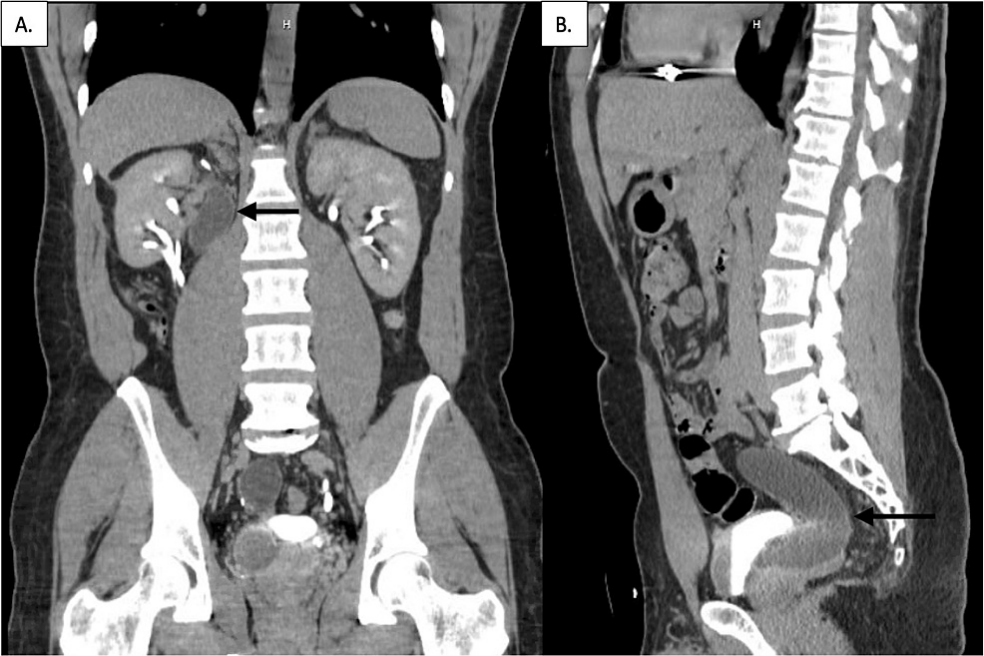
CT abdomen and pelvis with contrast three years post-operatively (A) Coronal view of the right kidney with resolution of hydronephrosis. Notice the dilated ureteral remnant adjacent to the kidney, marked by the black arrow. (B) Sagittal view of right ureter inserting into the posterior prostatic urethra demonstrating worsened hydronephrosis (black arrow).

He presented to the urology office three years later in 2022 with complaints of nausea and vomiting with minimal urinary symptoms and no flank pain. The patient’s creatinine had remained stable pre- and post-operatively at 0.9mg/dL and urinalysis at this time was negative. A CT urogram was obtained and the right system demonstrated two normal-sized ureters from the mid and lower poles of the kidney and a dilated ureteral remnant from the upper pole of the right kidney to the level of the bladder (Figure 2). The etiology of the patient’s nausea and vomiting was concluded to arise from his concurrent gastrointestinal conditions; he subsequently had an upper endoscopy performed confirming our suspicion.

## Discussion

The ontogeny of the kidney to the bladder connection is well described. The metanephros first appears in the fifth week of gestation which interacts with the ureteric bud to induce differentiation and form the functional units of the kidney [4]. The ureteric bud is derived from the caudal end of the mesonephric duct, also known as the Wolffian duct, which ultimately gives rise to the ureter. It is believed that a complete ureteral duplication and triplication arise from two or three independent ureteric buds, respectively, which give rise to separate ureters. While the Wolffian duct is migrating into the bladder, the ureteral buds undergo a switch resulting in the superior ureteral bud associating with the lower renal pole, while the inferior ureteral bud associates with the upper renal pole. During this rearrangement, the ectopic ureter will cross behind the orthotopic ureter which explains the Weigert-Meyer Law [4].

The Weigert-Meyer Law states in complete ureteral duplication the ureteral orifice draining the upper pole is usually medial and caudal, whereas the ureteral orifice correlating with the lower moiety is located lateral and cephalad. In an attempt to extend the Weigert-Meyer Law to triplicated systems, Zaontz and Maizels determined this principle applied to only 7 out of 13 patients with type I ureteral triplication [5]. Of patients with triplicated systems, Perkins described that the Weigert-Meyer law only applied to three of the eight patients studied [3]. As with our patient, the Weigert-Meyer Law cannot be applied reliably in ureteral triplication.

The unpredictability of ureteral orifice locations in relation to a triplicated system is theorized to be due to certain variabilities during nephrogenesis. The site of origin of the ureteric bud on the Wolffian duct may influence the point of insertion into the vesicourethral canal functionality of its associated renal unit. The distal end of the Wolffian duct can be divided into three segments, which migrate and become incorporated into the vesicourethral canal. Therefore, the location in which the ureteric buds arise along the Wolffian duct will determine the entry point into the vesicourethral canal [4]. Thus, in males, the more caudal the ureteric bud arises the more likely the ureter is to insert into the posterior urethra, as was the case with our patient.

Consequently, the occurrence of renal abnormalities is closely associated with the abnormal locations of that segment’s ureteral orifice, and when the orifice is displaced cranially or caudally, the ectopic segment shows more severe renal hypoplasia or dysplasia. The 14 patients with ureteral orifices located in the upper third of the posterior urethra or vas deferens included in the Mackie and Stephens study had non-functional renal segments consisting of minimal renal parenchyma associated with the ectopic ureter [4]. This appears to be consistent with our patient’s pathology after this respective renal segment was removed.

Although there have been no reported cases of obstructed prostatic megaureters in a type I triplicate system, there are several reports of obstructed duplicated ectopic ureters inserted into the prostatic urethra. In 1955, Ireland et al. reported a patient with a complete left triplication and a right ureteral duplication [6]. The upper moiety of the right-sided duplicated system was associated with an ectopic ureter terminating at the verumontanum. Due to long-standing obstruction, the upper pole moiety was dilated and non-functional, and a nephron-sparing approach was unable to be accomplished. While a ureteral remnant was left, this patient eventually underwent ureterectomy down to the level of the prostate after the remnant became infected. Similarly, Milicevic reports the case of a 32-year-old male with a duplicated system containing an ectopic ureter draining the upper pole of the left kidney and terminating in the posterior prostatic urethra [7]. Due to the dilation and non-functionality of the upper renal pole, this patient also underwent a partial nephrectomy and ureterectomy. Considering our patient's history of an abdominal gunshot wound with extensive abdominal adhesions, a ureterectomy was not carried out and the ureteral stump was left in place. Nonetheless, these cases offer excellent insight into the possible risks of leaving a ureteral remnant as well as ideal management in light of more favorable circumstances.

In regards to long-term outcomes when leaving a ureteral remnant behind, it is important to consider that vesicoureteral reflux and ureteral obstruction are the two most significant abnormalities seen in a duplicated or triplicated system. The presence of reflux in the setting of a ureteral stump may result in eventual dilation, a large diverticulum, or persistent infection. In the case of obstruction, the stump may be left open. It has further been described that a side-to-side anastomosis between the upper ureter and lower renal pelvis can be performed to relieve obstruction of the upper renal segment, assuming this segment is functional and not dilated [8]. Interestingly, Mcleod reports that a ureteroureterostomy can be effective regardless of a massively dilated and poorly functioning upper pole moiety, challenging the belief that the dysplastic segment must always be removed [9]. This of course applied to a pediatric population with a duplex system primarily, although this procedure was successful for the one child with a triplicated system included in the study.

A ureteroureterostomy is a common surgical technique for managing duplex collecting systems, but there is not much literature to support its use in a triplicated system. In 2022, the first account of a laparoscopic triple-ureteroureterostomy in a two-year-old girl with a completely triplicated system with dilated refluxing upper and middle renal moieties was published [10]. The location of all three ureteral orifices was identified prior to surgery, none of which were extravesicular. Because this procedure was successful in reducing hydronephrosis and vesicoureteral reflux of the affected segments, this begs the question of whether this can be a feasible surgical option in the adult population.

## Conclusions

Surgical options were limited for our patient given that the obstructed megaureter associated with the non-functional dilated upper pole was extravesicular. Ureteroureterostomies have been successful despite such massive dilation, but a concurrent taper and ureteral re-implantation would be necessary for the ectopic megaureter. Considering the function of the upper renal pole and extensive abdominal adhesions, this was not enough to justify proceeding with a more invasive procedure. While surgical management for adult patients with a completely triplicated system containing an obstructed non-functional renal unit is still not clearly defined, a partial nephrectomy with or without ureterectomy appears to be an appropriate course of management given the symptomatic relief of our patient without negatively impacting renal function.
